# Cuticular hydrocarbons corroborate the distinction between lowland and highland Natal fruit fly (Tephritidae, *Ceratitis rosa*) populations

**DOI:** 10.3897/zookeys.540.9619

**Published:** 2015-11-26

**Authors:** Lucie Vaníčková, Radka Břízová, Antonio Pompeiano, Sunday Ekesi, Marc De Meyer

**Affiliations:** 1 Laboratório de Ecologia Química, Instituto de Química e Biotecnologia, Universidade Federal de Alagoas, Maceió, AL, Brazil; 2 Institute of Organic Chemistry and Biochemistry ASCR, v.v.i., Flemingovo nám. 2, CZ-166 10 Prague 6, Czech Republic; 3 Institute of Chemical Technology in Prague, Technická 5, CZ-166 28 Prague 6, Czech Republic; 4 Laboratory of Plant Physiology, Center of Agricultural Sciences, Federal University of Alagoas, Maceió, Brazil; 5 International Center for Insect Physiology and Ecology, PO Box 30772-00100 GPO, Nairobi, Kenya; 6 Royal Museum for Central Africa, Leuvensesteenweg 13, B-3080 Tervuren, Belgium

**Keywords:** *Ceratitis rosa*, cryptic species, chemotaxonomy, GC×GC/MS, integrative taxonomy

## Abstract

The cuticular hydrocarbons (CHs) and morphology of two *Ceratitis rosa* Karsch (Diptera: Tephritidae) populations, putatively belonging to two cryptic taxa, were analysed. The chemical profiles were characterised by two-dimensional gas chromatography with mass spectrometric detection. CHs of *Ceratitis rosa* that originated from the lowlands and highlands of Kenya comprised of *n*-alkanes, monomethylalkanes, dimethylalkanes and unsaturated hydrocarbons in the range of the carbon backbone from C_14_ to C_37_. Hydrocarbons containing C_29_, C_31_, C_33_ and C_35_ carbon atoms predominated in these two populations. 2-Methyltriacontane was the predominant compound in both populations. Quantitative differences in the distribution of hydrocarbons of different chain lengths, mainly the C_22_, C_32_, C_33_ and C_34_ compounds of these two populations, were observed despite indistinct qualitative differences in these hydrocarbons. Morphological analyses of male legs confirmed that the flies belong to different morphotypes of *Ceratitis rosa* previously labelled as R1 and R2 for lowland and highland populations, respectively. A statistical analysis of the CH compositions of the putative R1 and R2 species showed distinct interspecific identities, with several CHs specific for each of the lowland and highland populations. This study supports a hypothesis that the taxon *Ceratitis rosa* consists of at least two biological species.

## Introduction

Sexual selection within populations can play an important role in speciation when divergence in mating signals and corresponding mate preference occur along different evolutionary trajectories in different populations (Jennings et al. 2014). In fruit flies (Diptera, Tephritidae), one potential target of sexual selection may be the blend of hydrophobic compounds on their cuticle, which often show intra- and interspecific variation, sexual dimorphism and may act as short-range pheromones (Carlson and Yocom 1986, Goh et al. 1993, Sutton and Carlson 1993, Vaníčková et al. 2012b, Vaníčková et al. 2014, Vaníčková et al. 2015). These compounds, cuticular hydrocarbons (CHs), play a major role in desiccation resistance, waterproofing, and/or mate choice, and may be under selection if particular components confer a mating advantage or increase the fitness of the resulting offspring (Howard and Blomquist 2005, Blomquist and Bagnères 2010, Gibbs 2011, Jennings et al. 2014). Characteristics of the CHs blend can vary with the diet, sex, age and geographic origin of a species and population (Blomquist and Bagnères 2010, Jennings et al. 2014).

In species of the fruit fly genus *Ceratitis*, courtship generally includes visual, auditory, tactile and olfactory cues (Shelly 2000, Aluja and Norrbom 2001, Yuval and Hendrichs 2001, Shelly et al. 2007). During courtship, male-borne volatiles are recognised in the initial phase and are detected by olfactory sensillae on the fly’s antennae, while less volatile compounds, such as CHs, may be exchanged during later courtship stages, when the male touches the female with legs and proboscis (Aluja and Norrbom 2001). The courted female chooses whether or not to mate with the male based on the quality of the various signals that he emits. Signal-preference co-evolution may provide mechanisms for both mate recognition and sexual selection in the early stages of population divergence, which may eventually lead to speciation (Jennings et al. 2014).

The Natal fruit fly, *Ceratitis rosa* Karsch (Diptera, Tephritidae), is a polyphagous species attacking a wide range of fruits on the African mainland. It has invaded some Indian Ocean islands, where it displaced the similarly introduced *Ceratitis capitata* (De Meyer 2000, De Meyer 2001, Duyck et al. 2004). *Ceratitis rosa*, together with *Ceratitis fasciventris* and *Ceratitis anonae*, are a closely related group of morphologically similar taxa known by researchers as the FAR species complex. The FAR complex has recently been studied by chemical, molecular genetic and morphological approaches to allow for discrimination of the putative species of this cryptic species complex (De Meyer 2001, De Meyer and Freidberg 2006, Virgilio et al. 2012, 2013, Vaníčková et al. 2014). Within the FAR complex, five genotypic groups have been identified and labeled as A (for *Ceratitis anonae*), F1 and F2 (for two *Ceratitis fasciventris* populations), and R1 and R2 (for two *Ceratitis rosa* populations) (Virgilio et al. 2013).

The chemical analyses of the cuticular hydrocarbon profiles of these putative species found significant differences between the A, F2 and R2 genotypes and characterised chemotaxonomic markers to distinguish these groups (Vaníčková et al. 2014). More recently, research has focused on the two *Ceratitis rosa* types, largely because *Ceratitis rosa* is considered the most economically important species within the complex (De Meyer 2001, Quilici et al. 2002, Baliraine et al. 2004). Adult males of the two *Ceratitis rosa* types can be differentiated based on the characters of the male, but not female, mid tibia (De Meyer et al. 2015); while analysis of wing land-marks using geometric morphometrics gives only a partial separation of the five FAR complex genotypes (Van Cann et al. 2015). Additional markers for the R1 and R2 populations are therefore needed.

The literature provides conflicting information regarding developmental physiology and climatic niche for *Ceratitis rosa*. Some studies indicate that *Ceratitis rosa* might be more tolerant of colder and wetter conditions than *Ceratitis capitata* (Duyck et al. 2004), suggesting greater potential for establishment in temperate regions (De Meyer et al. 2008). However, Grout and Stoltz (2007) indicate that *Ceratitis rosa* prefers hot and wet conditions. A re-analysis of the distributional data and historical material in collections shows that this might be because of the failure to differentiate between the two types (R1 and R2) that were indicated by the microsatellite study (Virgilio et al. 2013). R2 appears to occur at lower latitudes on the African continent and at higher altitudes – hereafter referred to as ‘highland’ type. It might be more cold resistant than the R1 type, which is absent from the colder parts (lower latitudes, higher altitudes) within the geographic range of *Ceratitis rosa* – hereafter referred to as ‘lowland’ or ‘coastal’ type (Tanga et al. 2015). The cold resistance may be directly connected to the cuticle composition as previously reported for other Diptera e.g. *Drosophila* sp. (Gibbs et al. 1997, Rouault et al. 2001, Rouault et al. 2004), and *Anopheles* sp. (Wagoner et al. 2014). With respect to CHs amount/*n*-alkane length, it is assumed that a reduction in water loss is the outcome of lower surface-area-to-volume ratio and reduced cuticle permeability, respectively (Rouault et al. 2004, Blomquist and Bagnères 2010, Gibbs 2011). Combining this background knowledge, leads us to hypothesize that CHs are likely to vary between R1 and R2 populations.

The purpose of the present study was, therefore, to identify the chemical constituents of the CHs and to analyse their variation between two populations of *Ceratitis rosa* (one highland and one lowland - based on morphological differentiation) originating from Kenya. These two populations were chosen for this study because they had previously been shown to be sexually incompatible (Ekesi et al. unpublished data), as well as having distinct male-borne volatile profiles (Kalinová et al. unpublished data). Additional to inter-population differences, we also evaluated sexual dimorphism in CHs composition within each population.

## Methods

### Insects

Pupae of two laboratory populations of *Ceratitis rosa* were obtained from the International Centre of Insect Physiology and Ecology (ICIPE, Nairobi, Kenya). The source colonies were established in 2012 and came from one lowland locality [Mwajamba, Msambweni, Coast Province, 04°18.21'S; 39°29.88'E, host fruit *Psidium guajava* (Myrtaceae), altitude 106 m, average temperature 28.1 °C] and one highland locality [Kithoka, Meru, Central Province, 00°05.59'N; 37°40.40'E, host fruit *Mangifera indica* (Anacardiaceae), altitude 1425 m, average temperature 21.5 °C] in Kenya (see Appendix). The pupae (F_2_ generation) were kept under identical laboratory conditions at the Institute of Organic Chemistry and Biochemistry (IOCB, Prague, Czech Republic). Flies were separated by sex within 24 hours of eclosion, fed on an artificial diet consisting of cane sugar and enzymatic yeast hydrolysate (in the ratio 3:1) and mineral water and kept at a relative humidity of 60%, at 25 °C, and a 12L:12D photoperiod.

### Chemical analyses

The extraction of the cuticular hydrocarbons of 20-day-old virgin males (*N* = 10) and females (*N* = 10) of the R1 and R2 morphotypes (resulting in *N* = 20 for R1 and *N* = 20 for R2) followed the methodology described in Vaníčková et al. (2012b) and Vaníčková et al. (2014). Flies were frozen at -18 °C and placed for 15 minutes into a desiccator to remove the surface moisture. In order to extract CHs from insect body surface individual fly was placed in small glass vials, which contained 0.5 mL of hexane (Fluka, Germany) and gently agitate for 5 minutes. 1-Bromdecane (Sigma-Aldrich, Czech Republic) was used as an internal standard for quantification (10 ng per 1 µL of the extract). Each extract was concentrated to approximately 100 µL by a constant flow of nitrogen and stored in a freezer (-5 °C) until analysis.

Two-dimensional gas chromatography with time-of-flight mass spectrometric detection (GC×GC/MS) was used for the quantification and identification of CH profiles. The analyses were performed on a LECO Pegasus 4D instrument (LECO Corp., St. Joseph, MI, USA) equipped with a non-moving quad-jet cryomodulator. A DB-5 column (J&W Scientific, Folsom, CA, USA; 30 m × 250 µm i.d. × 0.25 µm film) was used for GC in the first dimension. The second-dimension analysis was performed on a polar BPX-50 column (SGE Inc., Austin, TX, USA; 2 m × 100 µm i.d. × 0.1 µm film). Helium was used as a carrier gas at a constant flow of 1 mL min^-1^. The temperature program for the primary GC oven was as follows: 150 °C for 2 min, then 150–300 °C at 5 °C min^-1^, and finally a 10 min hold at 320 °C. The program in the secondary oven was 10 °C higher than in the primary one and was operated in an iso-ramping mode. The modulation period, the hot-pulse duration and the cool time between the stages were set to 3.0, 0.4 and 1.1 sec, respectively. The transfer line to the TOFMS was operated at 260 °C. The source temperature was 250 °C with a filament bias voltage of −70 eV. The data-acquisition rate was 100 Hz (scans/sec) for the mass range of 29–400 amu. The detector voltage was 1750V. For each sample, 1µL was injected in splitless mode. The inlet temperature was 200 °C. The purge time was 60 sec at a flow of 60 mL min^-1^. The data were processed and consecutively visualized on 2D and 3D chromatograms using LECO ChromaTOF^TM^ software. The *n*-alkane standard (C_8_–C_38_; Sigma-Aldrich) was co-injected with authentic samples to determine the retention indices (*RI*) of the analytes. The hydrocarbons were identified by a comparison of their mass spectra fragmentation patterns and *RI* (Van Den Dool and Kratz 1963, Carlson and Yocom 1986, Vaníčková 2012, Vaníčková et al. 2014).

### Morphological identification

Male specimens were shipped to the Royal Museum for Central Africa (RMCA), Tervuren, Belgium, where identifications were confirmed by M. De M. based on the pilosity and coloration of mid tibia (Virgilio et al. 2013, De Meyer et al. 2015).

### Statistics

The relative peak areas of 46 CH compounds (as identified by the GC×GC/MS in the deconvoluted total-ion chromatogram mode) were calculated in 10 replicate specimens for each sex of the two species (*N* = 40). Following Clarke (1993), we log-transformed the multivariate data in order to reduce the differences in scale between the variables while preserving information on the relative abundance of CHs across specimens.

A heat map was used to visualise the complex data sets organised as matrices. Heat maps make it possible to identify differences in the relative amounts of CHs between populations, with different compounds tending to form small clusters according to their quantities. To achieve this, the heat map performed two actions on a matrix of chromatographic peak areas. First, it reordered the rows and columns so that rows and columns with similar profiles were closer to one another, causing these profiles to be more visible to the eye. Second, each entry in the data matrix was displayed in a different colour, making it possible to view the patterns graphically. The dendrograms were created using correlation-based distances and the Ward method of agglomeration was applied in the present analysis (Key 2012).

To examine the differences between the two populations and sexes further, the percentage contribution of each compound to the average dissimilarity between the aforementioned factors was calculated with similarity percentage analysis (SIMPER) (Clarke 1993). All computations were performed with R 3.1.2 language and environment (R Core Team 2014) and the R packages *gplots* (Warnes et al. 2015) and *vegan* (Oksanen et al. 2015).

## Results

### CHs composition

**Figure 1.**
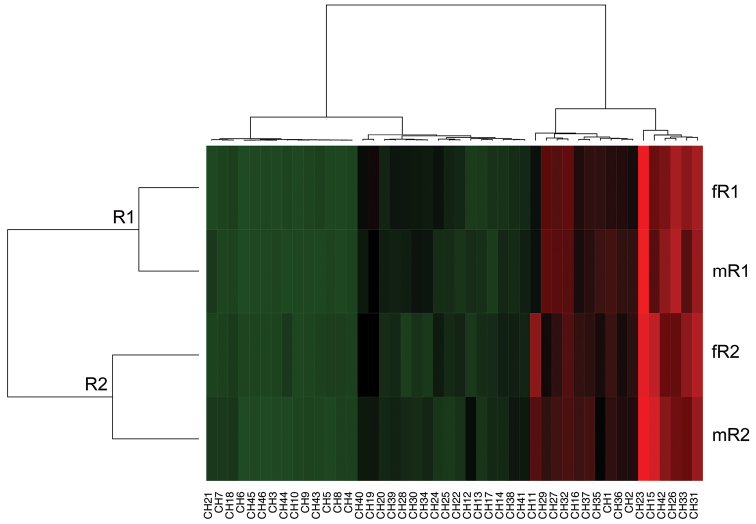
A heat map of the 46 cuticular hydrocarbons (columns, CH1-46) and the two *Ceratitis rosa* populations (rows, f-female, m-male) from the GC×GC/MS data set. The dendrograms are created using correlation-based distances and the Ward method of hierarchical clustering (*P* < 0.05). Putative morphotypes (R1 for the coastal population and R2 for the highland population) are depicted in the row dendrogram.

**Figure 2.**
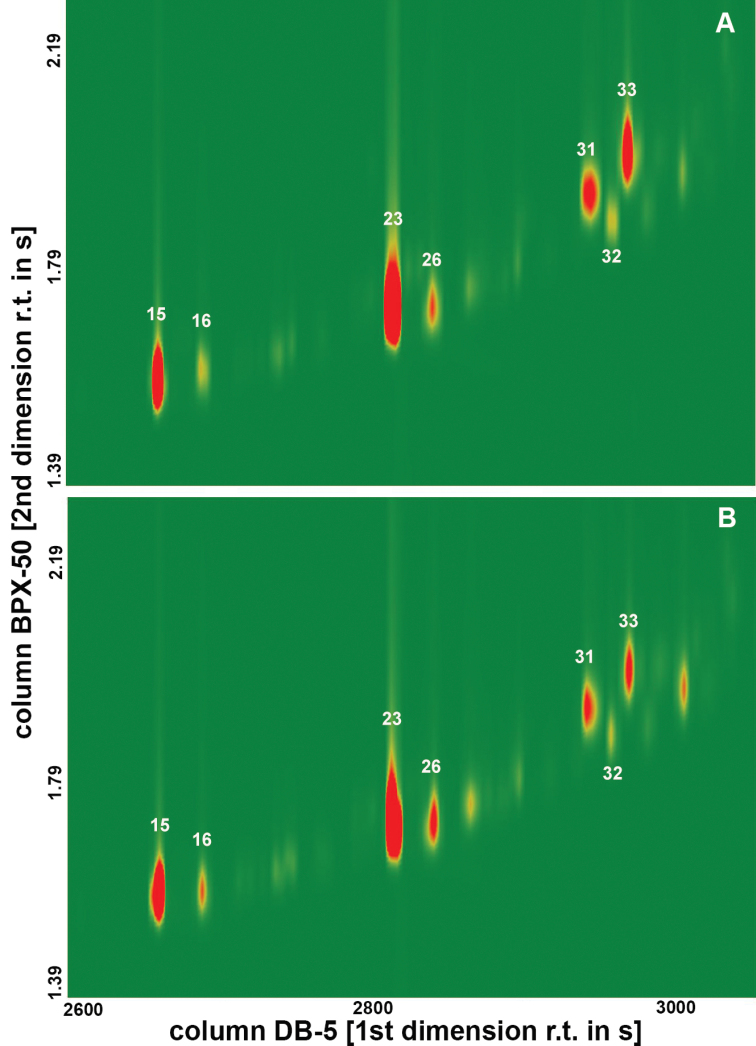
Section of the GC×GC/MS analysis of the female (**A**) and male (**B**) cuticular hydrocarbon profiles of the highland population (R2) of *Ceratitis rosa* from Kenya. The intensity of the signals is colour-coded from green (zero) to red (maximum). The compounds are assigned according to Table 1.

**Figure 3.**
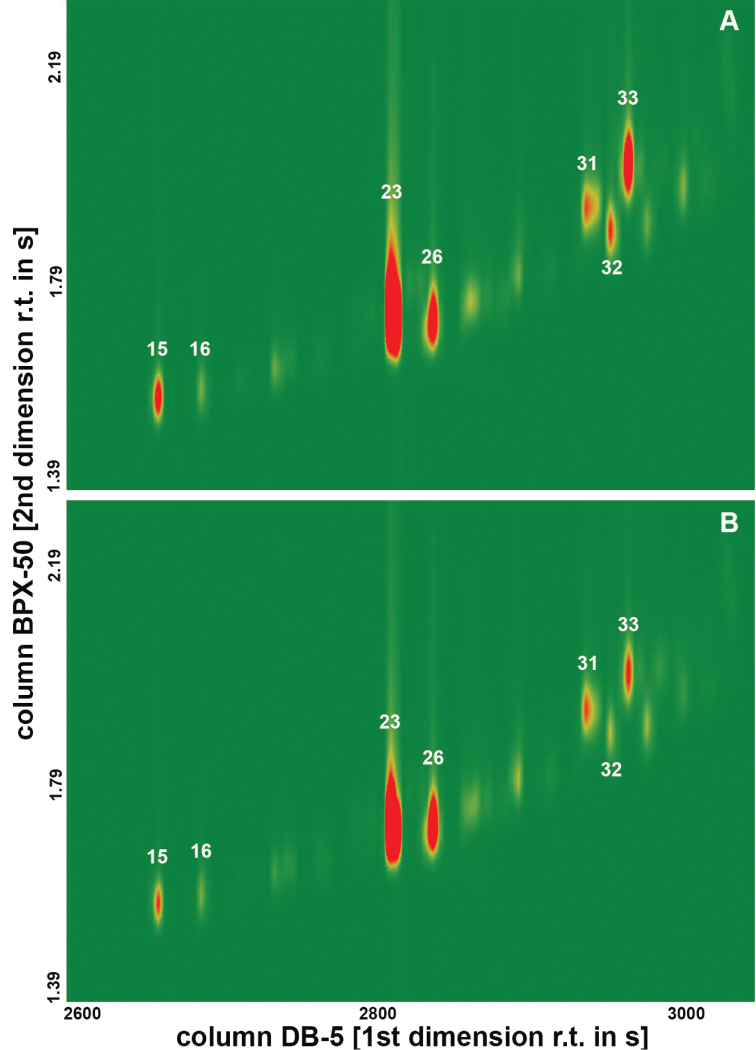
Section of the GC×GC/MS analysis of the female (**A**) and male (**B**) cuticular hydrocarbon profiles of coastal population (R1) of *Ceratitis rosa* from Kenya. The intensity of the signals is colour-coded from green (zero) to red (maximum). The compounds are assigned according to Table 1.

The GC×GC/MS analyses identified 46 peaks. The chain-length of the carbon backbones ranged from C_14_ to C_37_. The hydrocarbon profiles of the males and females included 5 *n*-alkanes, 19 methylbranched alkanes, 19 unsaturated alkanes, squalene, 1 aldehyde and 1 unidentified compound. The heat map characterised differences in the relative amounts of CHs between the *Ceratitis rosa* flies originating from highland and coastal regions (Figure 1). Marked quantitative differences were observed in the peaks between the two populations and genders. The most prominent peaks in all of the chromatograms were 2-methyltriacontane (2-MeC_30, _*RI 3064*, CH23) and tritriacontene (C_33:1_, *RI 3240*, CH31) (Figures 1–3).

**Table 1.** A comparison of the average abundance of important cuticular hydrocarbons between two morphotypes of *Ceratitis rosa* [coastal R1, highland R2]. The compounds are listed in the order of their contribution (δ_i_) to the average dissimilarity 5(δ_i_) between the two groups, with a cut-off when the cumulative percent contribution (∑δ_i_%) to δ_i _reaches 70%. The numbering of the compounds corresponds to Figure 1.

No.	Compound	*RI*	Abundance		δ_i_	δi /SD(δi)	% contr. diss.	∑δ_i_%
			R1 male	R2 male				
15	2-MeC_28_	2865	1.037	1.731	0.016	3.384	0.104	15
11	C_22:1_	2182	0.425	0.995	0.015	1.592	0.096	11
35	diMeC_31_	3297	0.883	0.447	0.010	2.884	0.065	35
26	diMeC_28_	3105	1.575	1.147	0.010	4.198	0.064	26
29	3-MeC_31_	3178	1.093	0.797	0.007	3.241	0.044	29
12	C_27:1_	2622	0.198	0.419	0.006	1.531	0.039	12
16	diMeC_26_	2902	0.613	0.869	0.006	2.443	0.039	16
30	diMeC_29_	3205	0.395	0.196	0.005	2.623	0.030	30
37	MeC_33_	3331	0.721	0.906	0.004	1.826	0.029	37
36	C_34:1_	3308	0.815	0.650	0.004	1.389	0.028	36
38	C_34:1_	3342	0.206	0.370	0.004	1.431	0.026	38
23	2-MeC_30_	3064	2.045	1.882	0.004	1.633	0.026	23
2	unknown	1402	0.816	0.649	0.004	2.035	0.025	2
27	7-/9-MeC_31_	3142	1.044	0.882	0.004	1.768	0.024	27
1	C_14_	1400	0.934	0.771	0.004	1.826	0.024	1
34	C_33:1_	3291	0.378	0.261	0.004	1.134	0.024	34
28	MeC_31_	3152	0.317	0.227	0.004	1.695	0.023	28
**No.**	**Compound**	***RI***	**Abundance**		**δ_i_**	**δi /SD(δi)**	% **contr.diss.**	∑**δ_i_**%
			**R1 female**	**R2 female**				
11	C_22:1_	2182	0.435	1.415	0.022	2.485	0.133	11
15	2-MeC_28_	2865	1.186	1.768	0.013	2.817	0.079	15
29	3-MeC_31_	3178	1.078	0.599	0.011	4.145	0.065	29
26	diMeC_28_	3105	1.534	1.191	0.008	2.284	0.047	26
34	C_33:1_	3291	0.352	0.112	0.007	1.789	0.044	34
28	MeC_31_	3152	0.384	0.066	0.007	3.160	0.043	28
33	C_33:1_	3280	1.353	1.444	0.005	1.337	0.029	33
30	diMeC_29_	3205	0.362	0.148	0.005	2.727	0.029	30
27	7-/9-MeC_31_	3142	1.043	0.835	0.005	1.875	0.029	27
36	C_34:1_	3308	0.705	0.639	0.005	1.373	0.029	36
1	C_14_	1400	0.732	0.925	0.005	1.791	0.028	1
42	C_35:2_	3460	1.263	1.223	0.004	1.381	0.027	42
2	unknown	1402	0.623	0.811	0.004	1.831	0.027	2
35	diMeC_31_	3297	0.803	0.629	0.004	1.384	0.026	35
16	diMeC_26_	2902	0.689	0.858	0.004	1.512	0.026	16
38	C_34:1_	3342	0.199	0.318	0.004	2.264	0.024	38
24	C_31:1_	3082	0.414	0.354	0.004	1.426	0.024	24
**No.**	**Compound**	***RI***	**Abundance**		**δ_i_**	**δi /SD(δi)**	% **contr. diss.**	∑**δ_i_**%
			**R1 male**	**R1 female**				
33	C_33:1_	3280	1.026	1.353	0.008	1.680	0.071	33
11	C_22:1_	2182	0.425	0.435	0.005	1.118	0.049	11
24	C_31:1_	3082	0.196	0.414	0.005	1.633	0.045	24
36	C_34:1_	3308	0.815	0.705	0.005	1.387	0.043	36
1	C_14_	1400	0.934	0.732	0.005	2.207	0.043	1
2	unknown	1402	0.816	0.623	0.004	2.217	0.041	2
15	2-MeC_28_	2865	1.037	1.186	0.004	1.220	0.040	15
40	C_34:2_	3371	0.311	0.242	0.004	1.376	0.034	40
42	C_35:2_	3460	1.352	1.263	0.004	1.500	0.034	42
34	C_33:1_	3291	0.378	0.352	0.003	1.160	0.031	34
35	diMeC_31_	3297	0.883	0.803	0.003	1.759	0.031	35
28	MeC_31_	3152	0.317	0.384	0.003	1.215	0.030	28
22	C_31:1_	3047	0.155	0.258	0.003	1.958	0.027	22
32	3-MeC_32_	3262	1.031	1.125	0.003	1.557	0.027	32
31	C_33:1_	3240	1.406	1.516	0.003	1.614	0.027	31
19	MeC_29_	2960	0.477	0.586	0.003	1.350	0.026	19
12	C_27:1_	2622	0.198	0.101	0.003	0.915	0.026	12
13	MeC_26_	2649	0.189	0.112	0.003	1.194	0.025	13
38	C_34:1_	3342	0.206	0.199	0.003	1.058	0.024	38
26	diMeC_28_	3105	1.575	1.534	0.003	1.439	0.024	26
16	diMeC_26_	2902	0.613	0.689	0.002	1.200	0.023	16
**No.**	**Compound**	***RI***	**Abundance**		**δ_i_**	**δi /SD(δi)**	% **contr. diss.**	∑**δ_i_**%
			**R2 male**	**R2 female**				
11	C_22:1_	2182	0.995	1.415	0.013	1.324	0.093	11
33	C_33:1_	3280	1.095	1.444	0.008	1.904	0.058	33
31	C_33:1_	3240	1.357	1.673	0.007	2.177	0.052	31
12	C_27:1_	2622	0.419	0.115	0.007	1.604	0.050	12
34	C_33:1_	3291	0.261	0.112	0.006	1.486	0.042	34
24	C_31:1_	3082	0.141	0.354	0.005	1.491	0.038	24
35	diMeC_31_	3297	0.447	0.629	0.005	1.451	0.033	35
29	3-MeC_31_	3178	0.797	0.599	0.005	2.054	0.033	29
42	C_35:2_	3460	1.275	1.223	0.004	1.211	0.030	42
2	unknown	1402	0.649	0.811	0.004	1.605	0.028	2
32	3-MeC_32_	3262	0.942	1.070	0.004	1.422	0.028	32
1	C_14_	1400	0.771	0.925	0.004	1.521	0.027	1
36	C_34:1_	3308	0.650	0.639	0.004	1.574	0.027	36
23	2-MeC_30_	3064	1.882	2.025	0.004	1.581	0.027	23
21	C_31:1_	3029	0.227	0.066	0.004	2.473	0.027	21
37	MeC_33_	3331	0.906	0.804	0.004	1.348	0.026	37
15	2-MeC_28_	2865	1.731	1.768	0.003	1.085	0.025	15
41	C_34:2_	3377	0.355	0.277	0.003	1.587	0.024	41
40	C_34:2_	3371	0.348	0.482	0.003	1.816	0.023	40
19	MeC_29_	2960	0.369	0.489	0.003	1.580	0.023	19

*RI* – retention index on the DB-5 column.

### Sexual dimorphism in CHs

The CH profiles of the virgin males and females differed qualitatively. SIMPER analyses, comparing conspecific males and females, revealed sex-specific compounds. In females the most abundant compounds were docosene (C_22:1_, *RI 2182*, CH11), hentriacontene (C_31:1_, *RI 3082*, CH24), 3-methyldotriacontane (3-MeC_32_, *RI 3272*, CH32) and tritriacontene (C_33:1_, *RI 3280*, CH33) (Table 1). In males, the compounds shared by coastal and highland flies were identified as tritriacontene (C_33:1_, *RI 3292*, CH34), tetratriacontene (C_34:1_, *RI 3308*, CH36) and pentatriacontadiene (C_35:2_, *RI 2416*, CH42) (Table 1). Interestingly, the compounds *n*-tetradecane (C_14_, *RI 1400*, CH1), unknown (*RI 1402*, CH2) and dimethylhentriacontane (diMeC_31_, *RI 3297*, CH35) were found to be specific for both coastal males and highland females (Table 1, Figure 1).

### Differences in the CH profiles between the highland and coastal *Ceratitis rosa*

Different patterns of CHs were detected between the two populations when constructing the heat map (Figure 1). The coastal (R1) population had higher amounts of dimethyloctacosane (diMeC_28_, *RI 3105*, CH26), 7-/9-methylhentriacontane (7-/9-MeC_31_, *RI 3142*, CH27), 3-methylhentriacontane (3-MeC_31_, *RI 3178*, CH29), 3-methyldotriacontane (3-MeC_32_, *RI 3272*, CH32) and pentatriacontadiene (C_35:2_, *RI 2416*, CH42), whereas the highland (R2) flies had higher amounts of docosene (C_22:1_, *RI 2182*, CH11), 2-methyloctacosane (2-MeC_28, _*RI 2865*, CH15) and dimethylhexacosane (diMeC26, *RI 2902*, CH16) on their cuticle. When the data were compared by SIMPER analyses, a pairwise comparison of the males or females between the two populations revealed the presence of two specific compounds that mostly contributed to the overall dissimilarity, suggesting these CHs to be potential chemotaxonomic markers. These compounds were identified as docosene (C_22:1_, *RI 2182*, CH11) and 2-methyloctacosane (2-MeC_28, _*RI 2865*, CH15) (Table 1, Figures 1–3).

## Discussion

Significant quantitative differences in the chemical CH profiles of the two populations of *Ceratitis rosa* have been demonstrated and complementary morphological analyses have confirmed that these two populations belong to two different morphotypes/genotypes, previously labelled by Virgilio et al. (2013) as R1 and R2.

The characteristic compounds of the lowland R1 type, diMeC_28_ and 3-MeC_31, _were present in higher relative amounts, whereas the highland R2 flies were characterised by high amounts of C_22:1_ and 2-MeC_28. _The compounds found in the present study correspond to the estimated chain lengths of the CH clusters identified in our earlier work for *Ceratitis rosa*, *Ceratitis anonae*, *Ceratitis fasciventris* and *Ceratitis capitata*, where the *Ceratitis rosa* R2 type could be determined based on the presence of even methylbranched hydrocarbons and the absence of odd methylbranched CHs when compared with the other three *Ceratitis* species (Vaníčková et al. 2014).

The intraspecific variation in the CH profiles between the two types reported here might be a result of several different factors, such as the effects of temperature, the social context and diet (Ferveur 2005, Kather and Martin 2012, Bontonou and Wicker-Thomats 2014, Vaníčková et al. 2015). Considering that the R2 type of *Ceratitis rosa* appears to be more cold resistant than the R1 type (Tanga et al. 2015), we assume that temperature may be one of the main sources of variation in R1 and R2 CH profiles. The coastal population of *Ceratitis rosa*, living at an average temperature of ~28 °C was characterised by greater amounts of long-chain CHs with carbon backbones from C_30_ to C_35_ when compared with the highland population living at an average temperature of ~21 °C. Long-chain CHs have higher melting points, which give them a superior capacity to limit water loss as compared to short-chain CHs (Bontonou and Wicker-Thomas 2014) and insect species or populations living in warmer, drier environments loose water less rapidly and have longer-chain CHs than mesic ones (Ferveur 2005). A recent study of six South American fruit fly populations has shown that the CH profile varies significantly with relative temperature, relative humidity and altitude (Vaníčková et al. 2015).

In *Ceratitis rosa*, we found that the differences in cuticular hydrocarbon profiles between the two populations were greater than those between the sexes, although there was still a significant quantitative sexual dimorphism. Our findings are in agreement with studies conducted on *Drosophila* sp., where differences between *Drosophila montana* populations were found to be considerably greater than those between the sexes (Veltsos et al. 2012, Jennings et al. 2014). Mating compatibility studies of the *Ceratitis rosa* flies from the same lowland and highland populations examined here have revealed a high degree of mating incompatibility between the two populations, where the index of sexual isolation (ISI) values ranged from 0.84 to 0.93, inferring reproductive isolation (Ekesi et al. unpublished data). The sex-specific differences in the quantitative composition of the *Ceratitis rosa* CH profiles identified in the present study indicate that these compounds might serve as short-range pheromones and thus could be directly involved in the mating compatibility/incompatibility within and between populations. Since the CHs involved in mating and courtship are not selectively neutral, reinforcing selection may cause closely related species to have distinct CH profiles (Blomquist and Bagnères 2010). A divergence in CH profiles between populations and sexes can lead to assortative mating and reproductive isolation, as shown in two populations of *Drosophila mojavensis* (Stennett and Etges 1997, Etges 1998). Studies on *Drosophila mojavensis* have demonstrated how even short-time isolation events can result in significant changes in CH composition (Stennett and Etges 1997, Etges 1998, Etges and Jackson 2001, Havens and Etges 2013).

It is important to note that the two populations of *Ceratitis rosa* studied here originate from different host plants, nevertheless they were reared during two generations on identical laboratory diet. The identified differences in the abundance of the CH between the populations and between the sexes may be, in addition to temperature and reproductive isolation factors, a result of the effects of host plants from which they originated (Stennett and Etges 1997, Vaníčková 2012, Vaníčková et al. 2012a). In *Drosophila* sp., the variation of CH profiles between closely related species of *Drosophila mojavensis* on varied cactus plants or between populations of these species reflects the adaptation to different host plants (Etges and Jackson 2001). The ratio of the principal CHs changed rapidly with laboratory acclimation and influenced courtship mating in *Drosophila mojavensis* (Stennett and Etges 1997). These CH changes depend on enzymes whose level could represent a metabolic adaptation to host-plant chemicals (Higa and Fuyama 1993, Jones 2001, Houot et al 2010). In tephritids, it is not known how are the CHs modified by diet composition and/or laboratory acclimation and whether any observed changes may impact the attractiveness of CH profiles. Therefore, future work needs to be conducted in order to elucidate the complex mechanisms involved in these events.

## Conclusion

Our data on cuticular hydrocarbon profiles, along with the previously published studies on morphology, genetics and sexual compatibility suggest that there exist two different entities, almost certainly unique biological species, within the taxa *Ceratitis rosa* from Kenya. In order to determine whether the different entities observed are consistent, the study needs to be extended to other populations of the two entities throughout their geographic and host ranges.

## Figures and Tables

**Figure:**
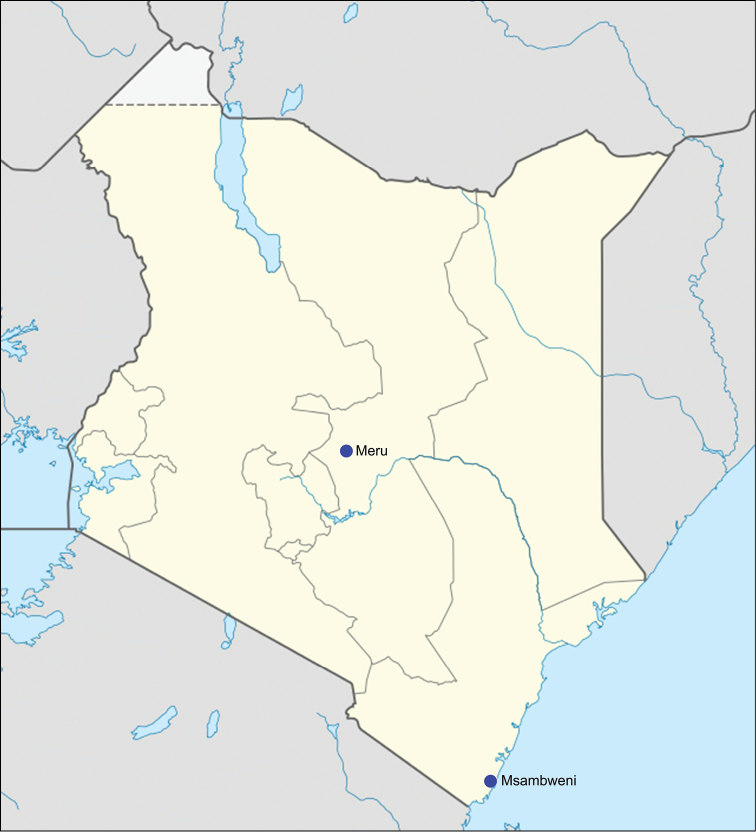
Map of the *Ceratitis rosa* collection area. Flies were picked up in Kenya, in Mwajamba, Msambweni (Coast Province) and in Kithoka, Meru (Central Province).

## References

[B1] Aluja M Norrbom AL (2001) Fruit Flies (Tephritidae): Phylogeny and Evolution of Behavior. CRC Press LLC, Boca Raton.

[B2] Baliraine FN Bonizzoni M Guglielmino CR Osir EO Lux SA Mulaa FJ Gomulski LM Zheng L Quilici S Gasperi G Malacrida AR (2004) Population genetics of the potentially invasive African fruit fly species, *Ceratitis rosa* and *Ceratitis fasciventris* (Diptera: Tephritidae). Molecular Ecology 13: 683–695. doi: 10.1046/j.1365-294X.2004.02105.x 14871371 10.1046/j.1365-294x.2004.02105.x

[B3] Blomquist GJ Bagnères AG (2010) Insect Hydrocarbons: Biology, Biochemistry, and Chemical Ecology. Cambridge University Press, New York. doi: 10.1017/CBO9780511711909

[B4] Bontonou G Wicker-Thomas C (2014) Sexual communiaction in the *Drosophila* genus. Insects 5: 439–458. doi: 10.3390/insects5020439 26462693 10.3390/insects5020439 PMC4592592

[B5] Carlson DA Yocom SR (1986) Cuticular hydrocarbons from six species of Tephritid fruit flies. Archives of Insect Biochemistry and Physiology 3: 397–412. doi: 10.1002/arch.940030407

[B6] Clarke KR (1993) Non-parametric multivariate analyses of changes in community structure. Australian Journal of Ecology 18: 117–143. doi: 10.1111/j.1442-9993.1993.tb00438.x

[B7] De Meyer M (2000) Phylogeny of the genus *Ceratitis* (Dacinae: Ceratitidini). In: Aluja M Norrbom AL (Eds) Fruit Fies (Tephritidae): Phylogeny and Evolution of Behavior. CRC, Boca Raton, 409–428.

[B8] De Meyer M (2001) On the identity of the Natal fruit fly *Ceratitis rosa* Karsch (Diptera, Tephritidae). Bulletin de l’Institut Royal des Sciences Naturelles de Belgique Entomologie 71: 55–62.

[B9] De Meyer M Freidberg A (2006) Revision of the subgenus *Ceratitis (Pterandrus)* Bezzi (Diptera: Tephritidae). Israel Journal of Entomology 35/36: 197–315.

[B10] De Meyer M Robertson MP Peterson AT Mansell MW (2008) Ecological niches and potential geographical distributions of Mediterranean fruit fly (*Ceratitis capitata*) and Natal fruit fly (*Ceratitis rosa*). Journal of Biogeography 35: 270–281.

[B11] De Meyer M Delatte H Ekesi S Jordaens K Kalinová B Manrakhan A Mwatawala M Steck G Van Cann J Vaníčková L Břízová R Virgilio M (2015) An integrative approach to unravel the *Ceratitis* FAR (Diptera, Tephritidae) cryptic species complex: a review. In: De Meyer M Clarke AR Vera MT Hendrichs J (Eds) Resolution of Cryptic Species Complexes of Tephritid Pests to Enhance SIT Application and Facilitate International Trade. ZooKeys 540: 405–427. doi: 10.3897/zookeys.540.10046 10.3897/zookeys.540.10046 PMC4714080 26798270

[B12] Duyck PF David P Quilici S (2004) A review of relationships between interspecifc competition and invasions in fruit flies (Diptera: Tephritidae). Ecological Entomology 29: 511–520. doi: 10.1111/j.0307-6946.2004.00638.x

[B13] Etges WJ (1998) Premating isolation is determined by larval rearing substrates in cactophilic *Drosophila mojavensis*. IV. Correlated responses in behavioral isolation to artificial selection on a life-history trait. American Naturalist 152: 129–144. doi: 10.1086/286154 10.1086/286154 18811406

[B14] Etges WJ Jackson LL (2001) Epicuticular hydrocarbon variation in *Drosophila mojavensis* cluster species. Journal of Chemical Ecology 27: 2125–2149. doi: 10.1023/A:1012203222876 11710615 10.1023/a:1012203222876

[B15] Ferveur JF (2005) Cuticular hydrocarbons: Their evolution and roles in *Drosophila pheromonal* communication. Behavior Genetics 35: 279–295. doi: 10.1007/s10519-005-3220-5 15864443 10.1007/s10519-005-3220-5

[B16] Gibbs A Chippindale A Rose M (1997) Physiological mechanisms of evolved desiccation resistance in *Drosophila melanogaster*. Journal of Experimental Biology 200: 1821–1832. 9225453 10.1242/jeb.200.12.1821

[B17] Gibbs AG (2011) Thermodynamics of cuticular transpiration. Journal of Insect Physiology 57: 1066–1069. doi: 10.1016/j.jinsphys.2011.05.003 21605563 10.1016/j.jinsphys.2011.05.003

[B18] Goh SH Ooi KE Chuah CH Yong HS Khoo SG Ong SH (1993) Cuticular hydrocarbons from two species of Malaysian *Bactrocera* fruit flies. Biochemical Systematics and Ecology 21: 215–226. doi: 10.1016/0305-1978(93)90039-T

[B19] Grout TG Stoltz KC (2007) Developmental rates at constant temperature of three economically important *Ceratitis* spp. (Diptera: Tephritidae) from Southern Africa. Environmental Entomology 36: 1310–1317. doi: 10.1603/0046-225X(2007)36[1310:DRACTO]2.0.CO;2 18284758 10.1603/0046-225x(2007)36[1310:dracto]2.0.co;2

[B20] Havens JA Etges WJ (2013) Premating isolation is determined by larval rearing substrates in cactophilic *Drosophila mojavensis*. IX. Host plant and population specific epicuticular hydrocarbon expression influences mate choice and sexual selection. Journal of Evolutionary Biology 26: 562–576. doi: 10.1111/jeb.12073 23286346 10.1111/jeb.12073

[B21] Higa I Fuyama Y (1993) Genetics of food preference in *Drosophila sechellia*. 1. Responses to food attractants. Genetica 88: 129–136. doi: 10.1007/BF02424469 8224853 10.1007/BF02424469

[B22] Houot B Svetec N Godoy-Herrera R Ferveur JF (2010) Effects of laboratory acclimation on the variation of reproduction-related characters in *Drosophila melanogaster*. 10.1242/jeb.041566 20543131

[B23] Howard RW Blomquist GJ (2005) Ecological, behavioral, and biochemical aspects of insect hydrocarbons. Annual Review of Entomology 50: 371–393. doi: 10.1146/annurev.ento.50.071803.130359 10.1146/annurev.ento.50.071803.130359 15355247

[B24] Jennings JH Etges WJ Schmitt T Hoikkala A (2014) Cuticular hydrocarbons of *Drosophila montana*: Geographic variation, sexual dimorphism and potential roles as pheromones. Journal of Insect Physiology 61: 16–24. doi: 10.1016/j.jinsphys.2013.12.004 24373710 10.1016/j.jinsphys.2013.12.004

[B25] Jones CD (2001) The genetica basis of larval resistance to a host plant toxin in *Drosophila sechellia*. Genetic Research 78: 225–233. doi: 10.1017/S0016672301005298 10.1017/s0016672301005298 11865712

[B26] Kather R Martin SJ (2012) Cuticular hydrocarbon profiles as a taxonomic tool: advantages, limitations and technical aspects. Physiological Entomology 37: 25–32. doi: 10.1111/j.1365-3032.2011.00826.x

[B27] Key M (2012) A tutorial in displaying mass spectrometry-based proteomic data using heat maps. BMC Bioinformatics 13 Suppl 16: S10. doi: 10.1186/1471-2105-13-S16-S10 10.1186/1471-2105-13-S16-S10 PMC3489527 23176119

[B28] Milet-Pinheiro P Navarro DMA De Aquino NC Ferreira LL Tavares RF Da Silva RCC Lima-Mendonça A Vaníčková L Mendonça AL Do Nascimento RR (2014) Identification of male-borne attractants in *Anastrepha fraterculus* (Diptera: Tephritidae). Chemoecology. doi: 10.1007/s00049-014-0180-3

[B29] Oksanen J Blanchet FG Kindt R Legendre P Minchin PR O’Hara RB Simpson GL Solymos P Stevens MHH Wagner H (2015) vegan: Community Ecology Package. R package version 2.2-1. http://CRAN.R-project.org/package=vegan

[B30] Quilici S Franck A Peppuy A Dos Reis Correia E Mouniama C Blard F (2002) Comparative studies of courtship behavior of *Ceratitis* spp. (Diptera: Tephritidae) in Reunion island. Florida Entomologist 85: 138–142. doi: 10.1653/0015-4040(2002)085[0138:CSOCBO]2.0.CO;2

[B31] R Core Team (2014) R: A language and environment for statistical computing. R Foundation for Statistical Computing, Vienna, Austria. http://www.R-project.org/

[B32] Rouault J Capy P Jallon J-M (2001) Variations of male cuticular hydrocarbons with geoclimatic variables: An adaptative mechanism in *Drosophila melanogaster*? Genetica 110: 117–130. doi: 10.1023/A:1017987220814 10.1023/a:1017987220814 11678502

[B33] Rouault JD Marican C Wicker-Thomas C Jallon JM (2004) Relations between cuticular hydrocarbon (HC) polymorphism, resistance against desiccation and breeding temperature; a model for HC evolution in *D. melanogaster* and *D. simulans*. Genetica 120: 195–212. doi: 10.1023/B:GENE.0000017641.75820.49 15088658 10.1023/b:gene.0000017641.75820.49

[B34] Shelly TE (2000) Male signalling and lek attractiveness in the Mediterranean fruit fly. Animal Behaviour 60: 245–251. doi: 10.1006/anbe.2000.1470 10973727 10.1006/anbe.2000.1470

[B35] Shelly TE Edu J Pahio E Nishimoto J (2007) Scented males and choosy females: does male odor influence female mate choice in the Mediterranean fruit fly? Journal of Chemical Ecology 33: 2308–2324. doi: 10.1007/s10886-007-9394-y 10.1007/s10886-007-9394-y 18030532

[B36] Stennett MD Etges WJ (1997) Premating isolation is determined by larval rearing substrates in cactophilic *Drosophila mojavensis*. III. Epicuticular hydrocarbon variation is determined by use of different host plants in *Drosophila mojavensis* and *Drosophila arizonae*. Journal of Chemical Ecology 23: 2803–2824. doi: 10.1023/A:1022519228346

[B37] Sutton BD Carlson BD (1993) Interspecific variation in Tephritid fruit fly larvae surface hydrocarbons. Archives of Insect Biochemistry and Physiology 23: 53–65. doi: 10.1002/arch.940230202

[B38] Tanga CM Manrakhan A Daneel JH Mohamed SA Khamis FM Ekesi S (2015) Comparative analysis of development and survival of two Natal fruit fly *Ceratitis rosa* Karsch (Diptera, Tephritidae) populations from Kenya and South Africa. In: De Meyer M Clarke AR Vera MT Hendrichs J (Eds) Resolution of Cryptic Species Complexes of Tephritid Pests to Enhance SIT Application and Facilitate International Trade. ZooKeys 540: 467–487. doi: 10.3897/zookeys.540.9906 10.3897/zookeys.540.9906 PMC4714083 26798273

[B39] Van Cann J Virgilio M Jordaens K De Meyer M (2015) Wing morphometrics as a possible tool for the diagnosis of the *Ceratitis fasciventris*, *C. anonae*, *C. rosa* complex (Diptera, Tephritidae). In: De Meyer M Clarke AR Vera MT Hendrichs J (Eds) Resolution of Cryptic Species Complexes of Tephritid Pests to Enhance SIT Application and Facilitate International Trade. ZooKeys 540: 489–506. doi: 10.3897/zookeys.540.9724 10.3897/zookeys.540.9724 PMC4714084 26798274

[B40] Van Den Dool H Kratz PD (1963) A generalization of the retention index system including linear temperature programmed gas-liquid partition chromatography. Journal of Chromatography A 11: 463–471. doi: 10.1016/S0021-9673(01)80947-X 10.1016/s0021-9673(01)80947-x 14062605

[B41] Vaníčková L (2012) Chemical ecology of fruit flies: Genera *Ceratitis* and *Anastrepha*. PhD thesis, Institute of Chemical Technology, Prague, Czech Republic.

[B42] Vaníčková L Břízová R Mendonça AL Pompeiano A Do Nascimento RR (2015) Intraspecific variation of cuticular hydrocarbon profiles in *Anastrepha fraterculus* (Diatrea: Tephritidae) species complex. Journal of Applied Entomology. doi: 10.1111/jen.12204

[B43] Vaníčková L do Nascimento RR Hoskovec M Ježková Z Břízová R Tomčala A Kalinová B (2012a) Are the wild and laboratory insect populations different in semiochemical emission? The case of medfly sex pheromone. Journal of Agricultural and Food Chemistry 60: 7168–7176. doi: 10.1021/jf301474d 22741541 10.1021/jf301474d

[B44] Vaníčková L Svatoš A Kroiss J Kaltenpoth M Nascimento RR Hoskovec M Břízová R Kalinová B (2012b) Cuticular hydrocarbons of the South American fruit fly *Anastrepha fraterculus*: Variability with sex and age. Journal of Chemical Ecology 38: 1133–1142. doi: 10.1007/s10886-012-0177-8 22948785 10.1007/s10886-012-0177-8

[B45] Vaníčková L Virgilio M Tomčala A Břízová R Ekesi S Hoskovec M Kalinová B Do Nascimento RR De Meyer M (2014) Resolution of three cryptic agricultural pests (*Ceratitis fasciventris*, *C. anonae*, *C. rosa*, Diptera: Tephritidae) using cuticular hydrocarbon profiling. Bulletin of Entomological Research 104: 631–638. doi: 10.1017/S0007485314000406 24896539 10.1017/S0007485314000406

[B46] Veltsos P Wicker-Thomas C Butlin RK Hoikkala A Ritchie MG (2012) Sexual selection on song and cuticular hydrocarbons in two distinct populations of *Drosophila montana*. Ecology and Evolution 2: 80–94. doi: 10.1002/ece3.75 22408728 10.1002/ece3.75 PMC3297180

[B47] Virgilio M Delatte H Quilici S Backeljau T De Meyer M (2013) Cryptic diversity and gene flow among three African agricultural pests: *Ceratitis rosa*, *Ceratitis fasciventris* and *Ceratitis anonae* (Diptera, Tephritidae). Molecular Ecology 22: 2526–2539. doi: 10.1111/mec.12278 23506441 10.1111/mec.12278

[B48] Virgilio M Jordaens K Breman FC Backeljau T De Meyer M (2012) Identifying insects with incomplete DNA barcode libraries, African fruit flies (Diptera: Tephritidae) as a test case. PLoS ONE 7: e31581. doi: 10.1371/journal.pone.0031581 10.1371/journal.pone.0031581 PMC3281081 22359600

[B49] Wagoner KM Lehmann T Huestis DL Ehrmann BM Cech NB Wasseberg G (2014) Identification of morphological and chemical markers of dry- and wet-season conditions in female *Anopheles gambiae* mosquitoes. Parasites and Vectors 7: 1–13. doi: 10.1186/1756-3305-7-294 24970701 10.1186/1756-3305-7-294 PMC4099382

[B50] Warnes GR Bolker B Bonebakker L Gentleman R Liaw WHA Lumley T Maechler M Magnusson A Moeller S Schwartz M Venables B (2015) gplots: Various R Programming Tools for Plotting Data. R package version 2.16.0. http://CRAN.R-project.org/package=gplots

[B51] Yuval B Hendrichs J (2001) Behavior of flies in the genus *Ceratitis* (Dacinae: Ceratitidini). In: Aluja M, Norrbom AL (Eds) Fruit Flies (Tephritidae) Phylogeny and Evolution of Behavior. CRC Press LLC, Boca Raton.

